# Evaluation of the effects of the COVID‐19 pandemic on dentistry

**DOI:** 10.1002/cre2.466

**Published:** 2021-06-30

**Authors:** Osman Evren Çelik, İbrahim Hüseyin Cansever

**Affiliations:** ^1^ Department of Oral and Dental Health Davraz Yaşam Hospital Isparta Turkey; ^2^ Faculty of Economics and Administrative Sciences, Department of Health Management Süleyman Demirel University Isparta Turkey

**Keywords:** COVID‐19, dentistry, health policy, pandemic

## Abstract

**Objectives:**

The aim of this study is to evaluate dentists' working conditions and the policies implemented for dentistry during the COVID‐19 pandemic. In addition, effects of working in private practice or governmental practice in terms of pandemic are also evaluated in the manuscript.

**Methods:**

A questionnaire was prepared to elicit dentists' working conditions during the pandemic and analyze and evaluate the policies implemented for dentistry. The questionnaires were sent to the dentists registered in the Turkish Dental Association (TDA) via e‐mail, and collected between September 30, 2020, and October 20, 2020. Descriptive statistical methods, validity and reliability analysis, and regression analysis were applied for data analysis.

**Results:**

Seven hundred thirty‐four dentists registered in the Turkish Dental Association took part in the study. 47% of respondents examined five or fewer patients per day during the pandemic. Dentists working in private practice examine more patients per day during the pandemic. 80.8% of the respondents experienced anxiety while examining patients during the pandemic. While the dentist's anxiety level increased with increasing the number of patients examined per day (*β*: 0.399), it decreased with increasing the dentist's age (*β*: −0.065). Respondents were not satisfied with the pandemic's management, with the decisions taken regarding dentistry, and with the supports provided to the dentists. 85.8% of the respondents were concerned about their professional future, which is higher among dentists who work in governmental practice (*p* < 0.05, ANOVA).

**Conclusions:**

Increasing dentists' representation in the management of the pandemic and the future policy‐making process, taking steps for the future by creating planning processes will eliminate the uncertainties and dissatisfaction and ensure to be ready for new pandemics.

## 1 INTRODUCTION

Dental treatments involve procedures where the patient and the dentist are in close contact, the dentist and other personnel are often exposed to body fluids such as blood and saliva of the patients, and in some cases, high‐speed cutting and piercing tools are used. During the operation of these high‐speed devices, water is cooled, and therefore an extensive amount of aerosol is released into the environment. These aerosols are contaminated with the patient's blood and saliva (Cleveland et al., 2016; Szymańska, 2007). Studies have shown that the SARS‐CoV‐2 virus can survive up to 3 h in aerosols and up to a few days on surfaces such as metal, glass, and plastic (Kampf et al., 2020; Otter et al., 2016; van Doremalen et al., 2020). Dental treatments are among clinical applications with the highest risk of exposure to respiratory pathogens due to the potential risk of aerosol contamination (Beltrán‐Aguilar et al., 2021; Zhang & Zheng, 2020). Although studies are showing that this contamination risk has been reduced with a series of protective measures (personal protective measures, using rubber dam, vacuum, and electrostatic extraction of aerosols during dental procedures) that can be taken, there is a strong need for new studies to investigate the risk (Fink et al., 2020; Gallagher et al., 2020; Harrel & Molinari, 2004). On the other hand, up to now there is no research conducted if the aerosols generated during dental care lead to transmission of SARS‐CoV‐2 (Banakar et al., 2020; Epstein et al., 2021; Levit & Levit, 2021). Recently it is reported that fine inhalable particles with a diameter of ≤ 2.5 μm (PM2.5) act as virus transport agent (Nor et al., 2021). While there is no evidence of SARS‐CoV‐2 transmission in the dental care, the primary transmission of COVID‐19 is through respiratory droplets and aerosols containing the virus. Hence, there is a gap of knowledge of the risk of aerosols generated during dental care led to transmission of SARS‐CoV‐2, and there is a strong need for new studies to investigate the SARS‐CoV‐2 transmission through aerosols generated during dental care.

When the SARS‐CoV‐2 virus emerged in China, hospitals and dental clinics took severe measures (China National Health Commission, 2020). When the virus spread in many cities in China, only emergency cases were treated, and routine dental practices were suspended during the pandemic (Guo et al., 2020). World Health Organization (WHO), on the other hand, in the COVID‐19 provisional guide, has recommended additional special measures that can be taken for aerosol spreading besides standard measures in dental practices during the epidemic (World Health Organization, 2020). In Turkey, the Ministry of Health recommended postponing non‐urgent dental procedures as much as possible (Republic of Turkey Ministry of Health, 2020a). With the spread of the virus in many countries, similar measures have been taken (American Dental Association, 2020; Coulthard, 2020; Dominiak et al., 2020).

Although patients with infected pneumonia are the primary source of infection, asymptomatic cases may also play a critical role in the transmission (Meng et al., 2020; Rothe et al., 2020), and hence, special precautions should be taken for each patient in dental clinics during the pandemic (Asan et al., 2020; Freund, 2020; Rothe et al., 2020). These precautions include preparation of all staff and clinical environment before patient admission, usage of personal protective equipment (PPE), and so on, and measures during and after treatment (Peng et al., 2020).

Based on these considerations, dentistry is one of the risky professions during the pandemic and evaluating the dentists' working conditions and the policies implemented for dentistry are very important. The aim of this study is to evaluate dentists' working conditions, the policies implemented for dentistry during the COVID‐19 pandemic. In addition, effects of working in private practice or governmental practice in terms of pandemic are also evaluated in the manuscript.

## 2 METHOD

### 2.1 Study design

In this study, a questionnaire was prepared to elicit the working conditions of dentists during the COVID‐19 pandemic and to analyze and evaluate the policies implemented for dentistry during the pandemic. The study was approved by Süleyman Demirel University Ethics Committee (30.09.2020, 45/3). The questionnaires were sent to the dentists registered in the Turkish Dental Association (TDA) via e‐mail and collected between September 30, 2020, and October 20, 2020. Seven hundred thirty‐four dentists registered in the TDA from different cities of Turkey took part in the study. All questionnaires were conducted via e‐mail.

The questionnaire includes nine demographic questions, three questions about the working status of dentists during the pandemic, and 14 statements regarding policy, working conditions, and emotional state on a five‐point Likert scale (1 = strongly disagree, 2 = disagree, 3 = undecided, 4 = agree, 5 = strongly agree). All questions of the questionnaire are represented in Tables 1, 2, 3. The study was conducted following the Checklist for Reporting Results of Internet E‐Surveys (CHERRIES) guidelines (Eysenbach, 2004).

**TABLE 1:** Demographic characteristics of the respondents

Variable	Category	*N*	%	Variable	Category	*N*	%
Age group	20–30	186	25.3	Professional experience (years)	0–5	172	23.4
	31–40	221	30.1		6–10	96	13.1
	41–50	171	23.3		11–15	122	16.6
	51–60	97	13.2		16–20	86	11.7
	≥60	59	8.0		≥20	258	35.1
Gender	Woman	399	54.4	Primary occupation	Governmental practice	150	20.4
	Man	335	45.6		Dental school faculty	97	13.2
Marital status	Married	513	69.9		Private practice	487	66.3
	Single	221	30.1	Medical conditions^a^	No	571	77.8
Children	Yes	446	60.8		Hypertension	69	9.4
	No	288	39.2		Diabetes	31	4.2
Education	Bachelor's degree	499	68.0		Cardiac disease	10	1.3
					Asthma	25	3.4
	Doctoral degree	235	32.0		COPD	3	0.4
					Nephropathy	5	0.7
					Other	46	6.2

Abbreviations: %, percent ratio; *N*: number of the respondents.

^a^ This is a multi‐response variable.

**TABLE 2:** Dental services served by the respondents during the COVID‐19 pandemic

Variable	Category	*N*	%	Variable	Category	*N*	%
Work status	Routine dental healthcare delivery	84	11.4	Number of patients per day	1–2	171	23.3
	Dental healthcare delivery to fewer patients	213	29.0		3–5	174	23.7
	Only emergency treatment	315	42.9		6–10	160	21.8
					˃10	107	14.5
	No dental healthcare delivery	122	16.6		None	122	16.6
Dental services delivered	All routine dental healthcare delivery					145	19.8
	Only emergency treatment					443	60.3
	Redeployed to help the health service in the field					24	3.3
	No dental healthcare delivery					122	16.6

Abbreviations: %, percent ratio; *N*, number of the respondents.

**TABLE 3:** Questions presented with Likert chart

Statement	x̄	*SD*
I had enough personal protective equipment during the pandemic	3.95	1.314
I had difficulty in obtaining personal protective equipment during the pandemic	3.11	1.105
I think adequate precautions are taken regarding COVID‐19 in my working place	3.26	1.099
I felt anxiety and/or stress while serving dental treatments during the pandemic	4.30	1.207
I read academic studies (national or international) examining the relationship between dentistry and pandemics during the pandemic	3.44	0.974
I read the articles, circulars, guidelines, and algorithms published by the Ministry of Health and/or the unit I am affiliated with during the pandemic	3.83	0.909
I think I am sufficiently informed or directed by the unit I am affiliated with during the pandemic	3.07	1.079
I think the overall pandemic management of Turkey is effective during the pandemic	2.59	0.977
I think enough measures are taken for dentistry in Turkey during the pandemic	2.41	1.002
I know the supports offered by the government (soft loan, postponing insurance charges, extended declaration periods, short‐time working allowance, etc.) to dentists during the pandemic	3.14	1.085
I think the economic supports provided by the government to dentists are sufficient during the pandemic	2.02	0.924
My education in dentistry school was beneficial in terms of my professional behaviors during the pandemic	3.65	0.966
I was worried about my professional future during the pandemic	3.69	1.093
I am ready for new pandemics	2.66	0.960

Abbreviations: *SD*, standard deviation; x̄: mean.

### 2.2 Policy evaluation

Policies regarding dentistry in the COVID‐19 were also examined in the study. Policy evaluation is a study to measure the success of public policies to be implemented or are currently implemented (Gül & Acar, 2018). For this purpose, along with the findings of the questionnaires, active circulars, and the articles, recommendations, and guidelines by the Ministry of Health and TDA were taken into consideration.

### 2.3 Statistical analysis

Statistical Package for the Social Sciences v27.0 was used for the statistical evaluation of the data obtained in this study. Descriptive statistical methods, validity, and reliability analysis were applied for data analysis. Cronbach's alpha coefficient was used for reliability. The alpha coefficient was 0.714, suggesting that the data have acceptable internal consistency. Kurtosis and skewness were calculated to test normality and the values of which show that the data were normally distributed (Tabachnick & Fidell, 2006). Test of homogeneity of variance was performed and variances were checked to ensure homogeneity. One way complete statistical analysis of variance (ANOVA) test at a confidence level of 95% applied to the results of the questionnaire. Tukey test was utilized for determining the significant differences among the groups. The level of significance was set at 0.05.

## 3 RESULTS

With the rapidly spreading SARS‐CoV‐2 virus in the first months of 2020, all healthcare professionals have faced a great struggle. The risk of transmission of COVID‐19 to healthcare workers who are in close contact with patients is relatively high (Wang et al., 2020). There is a very high risk of COVID‐19 transmission to dentists due to face‐to‐face communication with patients, exposure to saliva, blood, and other body fluids, and using sharp tools in their routine (Ge et al., 2020). Dentists are more likely to be affected by this disease than other healthcare professionals (Gamio, 2020). The findings of the questionnaire prepared in this study give an insight into dentistry during the pandemic.

The respondents' demographic characteristics and the information about the dental services served by the respondents during the pandemic are presented in Tables 1 and 2, respectively. 22.2% of the respondents have chronic diseases and mostly reported chronic diseases are hypertension, diabetes, and asthma. 42.9% of the respondents served only emergency treatments during the pandemic. The rate of routine dental healthcare delivery during the pandemic is higher among dentists working in private practice. The respondents working in private practice served the highest number of patients, and the respondents working in faculties served the least number of patients during the pandemic. The findings of the questionnaire showed that respondents served mostly emergency treatments (60.3%), and the number of patients served by dentists reduced during the pandemic.

The distribution of all questions presented with the Likert chart is shown in Table 3. The average of the responses (x̄) to the phrase about having enough PPE during the pandemic is 3.95 (standard deviation (SD): 1.314), which is relatively high. The number of respondents working in governmental practice stating having enough PPE during the pandemic is higher than the respondents working in private practice (*p* < 0.05, ANOVA). Most of the respondents who stated having difficulty obtaining PPE during the pandemic are the respondents working in private practice (*p* < 0.05, ANOVA).

Most of the respondents stated feeling anxiety and/or stress while serving dental treatments during the pandemic (x̄: 4.30, SD: 1.207). According to the standardized regression analysis, the level of anxiety increased with the increasing number of the patients served (regression coefficient (*β*): 0.399), and the level of anxiety decreased with the increasing age (*β*: −0.065). Besides, while the anxiety level is the lowest among the respondents without chronic diseases, the anxiety level is the highest among the respondents with diabetes and asthma (*p* < 0.05, ANOVA).

The respondents read articles, circulars, guidelines, and algorithms published by the Ministry of Health and/or the unit they are affiliated with (x̄: 3.83, *SD*: 0.909) more than the academic studies (x̄: 3.44, *SD*: 0.974). While the highest number of the respondents reading articles, circulars, guidelines, and algorithms published by the Ministry of Health and/or the unit they are affiliated with are working in private practice (*p* < 0.05, ANOVA), the highest numbers of the respondents reading academic studies are female dentists who had a doctorate (*p* < 0.05, ANOVA).

The respondents are neutral about the sufficiency of the information or directions provided by the unit they are affiliated with during the pandemic (x̄: 3.07, *SD*: 1.079). The respondents working in private practice are more satisfied with the information or directions provided by the unit they are affiliated with during pandemic than the respondents working in governmental practice or dental school (*p* < 0.05, ANOVA). The information or directions were conveyed to the respondents by the units they are affiliated with, mostly via e‐mail and SMS.

The satisfaction of the respondents about the overall pandemic management (x̄: 2.59, *SD*: 0.977) and measures taken for dentistry during pandemic (x̄: 2.41, *SD*: 1.002) are low. The satisfaction level of the respondents about the measures taken for dentistry during the pandemic is the lowest among the respondents working in governmental practice (*p* < 0.05, ANOVA). The respondents stated that the economic supports provided by the government to dentists are not sufficient (x̄: 2.02, *SD*: 0.924). The respondents who read the articles, circulars, guidelines, and algorithms published by the Ministry of Health and/or the unit they are affiliated with are more satisfied with the measures are taken for dentistry during the pandemic (*p* < 0.05, ANOVA). Besides, respondents working in the private practice, elderly, and with high professional experience are more satisfied with the measures taken for dentistry during the pandemic (*p* < 0.05, ANOVA).

Even though the respondents stated that their dentistry school education was beneficial for their professional behaviors during the pandemic (x̄: 3.65, *SD*: 0.966), they do not feel ready for new pandemics (x̄: 2.66, *SD*: 0.960). Most of the respondents are concerned about their professional future (x̄: 3.69, *SD*: 1.093). Respondents working in private practice are more concerned about their professional future. Moreover, respondents working in private practice, elderly, and high professional experience are more prepared for new pandemics (*p* < 0.05, ANOVA).

## 4 DISCUSSION

The aim of this study is to evaluate dentists' working conditions, the policies implemented for dentistry during the COVID‐19 pandemic.

### 4.1 Healthcare services

The American Dental Association recommended postponing all dental procedures, except in emergencies (American Dental Association, 2020). While decisions were taken to close almost all dental clinics in Singapore, Taiwan, and Hong Kong, decisions were taken to continue conducting routine treatment of patients who did not have close contact with COVID‐19 patients initially in the UK (Coulthard, 2020). In Turkey, the rules to be followed in dental treatments and the emergency and compulsory dentistry services have been defined, with the regulations prepared by the Coronavirus Scientific Committee and the Ministry of Health (Republic of Turkey Ministry of Health, 2020b, 2020c). It was proposed to cancel all appointments and create new appointment schedules with the circular prepared by the Ministry of Health (Republic of Turkey Ministry of Health, 2020d). Detailed algorithms have been created by the TDA scientific board regarding the triage procedures to be applied in emergencies (Turkish Dental Association, 2020a). The conditions to be provided in the private practice clinics providing oral and dental health services during and after the pandemic have been explained in detail (Turkish Dental Association, 2020b). Similar to TDA, the Polish Dental Association published recommendations on dental treatments during pandemic. They recommended conducting only emergency treatments during the pandemic (Dominiak et al., 2020). The Polish Ministry of Health required the implementation of enhanced PPE for aerosol‐generating procedures, such as filtering facepiece respirators, disposable fluid‐resistant gowns, airtight eye protection, and full‐face shields (Tysiąc‐Miśta & Dziedzic, 2020).

In Turkey, dentists work in governmental practice, dentistry schools, or private practice. The total number of dentists working in Turkey in 2018 was 30.635. 10.814 of them were working in governmental practice, 4.244 of them in dentistry schools, and 15.577 of them in private practice (Ministry of Health, 2019). It can be seen that more than half the dentists are working in private practice in Turkey. In addition, the number of patients served dental treatment was 53.115.784 in 2018, and the rate of dental treatment per capita was 0.65 (Ministry of Health, 2019). The decisions made by the Ministry of Health are circulated to the private practice dentists by the TDA. The respondents working in private practice are the most satisfied group about information or directions provided by the unit they are affiliated with during the pandemic. TDA actively reaches dentists working in private practice, ensuring more effective implementation of the decisions.

During the pandemic, only emergency and mandatory services were given with fewer personnel in public hospitals (Republic of Turkey Ministry of Health, 2020a). However, there was no such implementation for private practice dentists. Some of the private practice dentists kept working, as usual, some of them reduced the number of patients treated per day, or some did not work during the pandemic. A study conducted in Poland revealed that 31% of the dentists had to kept working during pandemic due to their financial situations, and 15.5% due to the instructions of their employer (Tysiąc‐Miśta & Dziedzic, 2020). If the economic supports provided by the government are planned for the necessities of the dentists, private practice dentists will also follow the recommendations of the Ministry of Health of serving only emergency treatments.

### 4.2 Emotional status

Healthcare workers are at high risk of infection as they are more likely to be directly exposed to the virus, causing fear and anxiety in healthcare workers. It has been found that healthcare workers had high levels of fear and anxiety during SARS in Canada (Robertson et al., 2004). Similarly, the respondents have a high level of fear and anxiety during the pandemic. The source of stress/anxiety in healthcare workers is difficulties obtaining PPE, insufficient PPE, limited testing and treatment options, and lack of knowledge on the disease (Mattos & Pordeus, 2020). Most of the participants of this study stated feeling anxiety and/or stress while serving dental treatments during the pandemic. The level of anxiety increased with the increasing number of the patients served. In a study conducted in Poland, it was revealed that dentists felt fear and anxiety while serving dental treatments (Tysiąc‐Miśta & Dziedzic, 2020). In another study in Italy, it was shown that dentists felt fear of COVID‐19 transmission while serving dental treatments (Consolo et al., 2020).

It is very important to take strict measures against the pandemic in dentistry, review the guidelines and protocols prepared about the pandemic in dentistry, deliver these guidelines and protocols to all dentists, and ensure the implementation of the protocols. All these precautions will be useful in reducing the anxiety/stress on dental professionals.

### 4.3 Economy

COVID‐19 is one of the most critical crises of this century in the world (Aydın, 2020). During the pandemic, many economic and social difficulties have emerged (Alkan, 2020). Various restrictions were brought into life all over the world. The measures taken to prevent the spread of the virus have affected all sectors economically, including dental clinics (Farooq & Ali, 2020). Schwendicke et al., conducted an economic analysis of the COVID‐19 on dental practices. They demonstrated the severity of the economic effects of the pandemic related policies on dental practices (Schwendickea et al., 2020). The respondents of our research are not satisfied with the economic supports provided by the government to dentists. It is crucial to increase the scope of the economic support provided by taking the suggestions of the relevant institutions.

### 4.4 Policies

The COVID‐19 pandemic has caused many problems in the dental health system due to inadequate coordination of pandemic‐related services on a global scale (Tysiąc‐Miśta & Dziedzic, 2020). According to the findings of a questionnaire conducted in May 2020, 73.7% of the respondents were satisfied with the overall pandemic management (Anar, 2020). However, the respondents of our research were not satisfied with the overall pandemic management and the measures taken for dentistry during the pandemic. These findings show that the target group of the questionnaires is very effective in the results.

Dental associations in various countries including the American Dental Association (Quiñonez & Vujicic, 2020), European countries (Consolo et al., 2020; Tysiąc‐Miśta & Dziedzic, 2020; Poland, Italy and Germany, etc.), and others (Faccini et al., 2020; Schwendickea et al., 2020) have various actions related to dental service during pandemic. At the beginning of the pandemic, it was advised to postpone non‐emergency dental procedures until the pandemic gets better. The result s of a study conducted by the American Dental Association Health Policy Institute showed that at the beginning of the pandemic most of the dental clinics in USA were closed (79.4%) but seeing emergency patients only. However, in April 2021 more than half of the clinics (58.1%) were started working as usual, and the rest (41.5%) are working lower patient volume than usual. The number of the clinics which are still closed is almost negligible (less than 0.5%). In the same study respondents stated that they raised fee (21.6%), borrowed money from bank (15.9%), or changed the material supplier (15.5%) to maintain the financial sustainability of their clinics. Only 1.8% of the respondents stated using government loans (American Dental Association, n.d.).

Involving stakeholders in the policy‐making process ensures the process to be more effective, and solves the problems, and is more successful in implementation (Kilpatrick, 2009). Although it is advantageous to have dentists on the pandemic science board, including representatives from professional associations and non‐governmental organizations in the pandemic management process will be beneficial in managing the process more effectively.

WHO World Health Assembly established an integrated action plan for promoting dental health and preventing dental diseases in 2007 (Petersen et al., 2020). Prevention measures for dentistry from the COVID‐19 pandemic or new pandemics should be included in these action plans immediately.

The SARS‐CoV‐2 virus has a very high transmission rate. Dental procedures have the potential to increase the possibility of transmission of the SARS‐CoV‐2 virus. Dentists mentioned feeling anxiety/stress while serving dental treatments during the pandemic not only in Turkey but also in other countries, for example, Poland. All countries have implemented various actions related to dental service during the pandemic. At the beginning of the pandemic, in most of the countries the dental clinics were closed but seeing emergency patients only. However, because of the financial reasons dental clinics are starting working as usual in many countries like Turkey, USA, and so on. Not only in Turkey but also in USA the governmental supports to the dental clinics is very limited during the pandemic. Many countries required the usage of PPE while dental treatments, but some countries like Poland implementation of enhanced PPE for aerosol‐generating procedures is required.

## 5 CONCLUSIONS

This study shows that dentists are not satisfied with the overall management of the pandemic, measures taken for dentistry during the pandemic, and economic supports provided by the government to dentists. Dentists experienced anxiety and/or stress while serving dental treatments during the pandemic, and they are concerned about their professional future. To prevent this dissatisfaction and concerns, it is necessary to increase the representation of dentists in the dental policy‐making process, increase the government supports to dentists, and make necessary plans for new pandemics. From this work, a fundamental understanding of the effects of the pandemic on dental professionals, who are among the highest risk group of the COVID‐19 transmission, can be achieved. Research similar to this study needs to be conducted in other countries to better evaluate the effects of the COVID‐19 pandemic on dentistry.

## CONFLICT OF INTEREST

No potential conflicts of interest regarding the publication of this manuscript.

## Data Availability

The data that support the findings of this study are available from the corresponding author upon reasonable request.
